# Memory impairment in the D2.*mdx* mouse model of Duchenne muscular dystrophy is prevented by the adiponectin receptor agonist ALY688

**DOI:** 10.1113/EP091274

**Published:** 2023-07-06

**Authors:** Catherine A. Bellissimo, Laura N. Castellani, Michael S. Finch, Mayoorey Murugathasan, Shivam Gandhi, Gary Sweeney, Ali A. Abdul‐Sater, Rebecca E. K. MacPherson, Christopher G. R. Perry

**Affiliations:** ^1^ School of Kinesiology & Health Science York University Toronto ON Canada; ^2^ Muscle Health Research Centre York University Toronto Canada; ^3^ Department of Health Sciences Brock University St Catharines ON Canada; ^4^ Department of Biology York University Toronto Ontario Canada

**Keywords:** cognition, Duchenne muscular dystrophy, memory, mitochondria

## Abstract

Memory impairments have been well documented in people with Duchenne muscular dystrophy (DMD). However, the underlying mechanisms are poorly understood, and there is an unmet need to develop new therapies to treat this condition. Using a novel object recognition test, we show that recognition memory impairments in D2.*mdx* mice are completely prevented by daily treatment with the new adiponectin receptor agonist ALY688 from day 7 to 28 of age. In comparison to age‐matched wild‐type mice, untreated D2.*mdx* mice demonstrated lower hippocampal mitochondrial respiration (carbohydrate substrate), greater serum interleukin‐6 cytokine content and greater hippocampal total tau and Raptor protein contents. Each of these measures was partly or fully preserved after treatment with ALY688. Collectively, these results indicate that adiponectin receptor agonism improves recognition memory in young D2.*mdx* mice.

## Abstract (highlights)

- **What is the central question of this study?**
  Can adiponectin receptor agonism improve recognition memory in a mouse model of Duchenne muscular dystrophy?
- **What is the main finding and its importance?**
  Short‐term treatment with the new adiponectin receptor agonist ALY688 improves recognition memory in D2.*mdx* mice. This finding suggests that further investigation into adiponectin receptor agonism is warranted, given that there remains an unmet need for clinical approaches to treat this cognitive dysfunction in people with Duchenne muscular dystrophy.

## 1 INTRODUCTION

Duchenne muscular dystrophy (DMD) is a severe, progressive muscle‐wasting disorder that results from mutations to the gene for the protein dystrophin (Bulfield et al., 1984; Emery, 1993; Hoffman, 2020). By compromising cell membrane stability and cytoskeletal architecture, a variety of cell stressors arise, including metabolic dysfunction, which might contribute to muscle weakness (reviewed by Bellissimo et al., 2022; Guiraud & Davies, 2017). Dystrophin is also expressed in the CNS, including the hippocampus and other regions that regulate memory processing (Doorenweerd et al., 2017).

Previous reports estimate that ∼30% of people with DMD exhibit cognitive dysfunction, including memory impairments (Rae & O'Malley, 2016; Snow et al., 2013). Similar observations have been reported in murine models of DMD, including C57BL/10ScSn‐*mdx* and D2.*mdx* mice (Hayward et al., 2022; Vaillend et al., 2004). Such cognitive impairments might be related to disruptions in brain energy homeostasis, given that separate investigations using non‐invasive magnetic resonance spectroscopy reported elevated inorganic phosphate (P_i_) to ATP and P_i_ to phosphocreatine (PCr) in boys with DMD (Tracey et al., 1995). Similar observations were reported in C57BL/10ScSn‐*mdx* mice (Tracey et al., 1996), who found that maximal activities of mitochondrial enzymes in the brain were not different from wild‐type (WT) mice, suggesting that the capacity of specific mitochondrial pathways might be preserved. However, functional assessments of oxidative phosphorylation using intact mitochondria have not been performed. Such approaches have revealed attenuations in mitochondrial substrate oxidation in muscle from *mdx* mice and muscle from people with DMD (reviewed by Bellissimo et al., 2022).

Glucocorticoids (GCs) are the current standard of care for slowing the progression of muscle weakness in males with DMD (Balaban et al., 2005; DeSilva et al., 1987; Marden et al., 2020) by reducing inflammation (Kim et al., 2015). The effects of GCs on memory in DMD are not fully resolved in the literature, but memory impairments attributed to GC therapy have been identified more broadly in other populations (reviewed by Brown et al., 2007). As such, there is an unmet need to develop new therapies that improve memory in addition to muscle function in DMD. In this regard, adiponectin receptor (AdipoR) agonism was recently shown to improve markers of muscle quality in *mdx* mice (Abou‐Samra et al., 2020). The agonist ALY688 (also referred to as ADP355; Otvos et al., 2011), in particular, attenuates inflammation in various inflammatory disorders, such as dry eye and liver diseases (Kumar et al., 2014; Wang et al., 2016), and reprograms substrate metabolism (Da Eira et al., 2020). Given that adiponectin‐deficient mice have impaired recognition memory (Bloemer et al., 2019), we hypothesized that a recently developed slow‐release formulation (ALY688‐SR) with improved pharmacokinetic properties enabling once‐daily injection (Allysta Pharmaceutical, unpublished observations) would improve memory during early disease stages in young D2.*mdx* mice, in association with lower inflammation and enhanced mitochondrial metabolism.

## 2 MATERIALS AND METHODS

### 2.1 Animals

Male D2.*mdx* mice originated from a colony maintained at York University (Toronto, ON, Canada) and were sourced from Jackson Laboratories (stock number 013141; Bar Harbor, ME, USA). Mice were treated daily from day 7 to 28 of age with ALY688‐SR at 15 mg/kg body weight (see the Acknowledgments for more information; from Allysta Pharmaceuticals, Bellevue, WA, USA; D2.*mdx*‐ALY688‐SR) or saline control (D2.*mdx*‐VEH). This age was chosen because it captures an early stage of disease progression in cardiac and skeletal muscle (Bellissimo et al., 2023; Coley et al., 2016; Hughes et al., 2020, 2019; Ramos et al., 2020). Mice were killed 20–24 h after the last dose. Breeding of WT mice was unsuccessful, similar to a previous report (Holmes, 2003). Instead, 3‐week‐old WT DBA/2J WT mice (D2A) were obtained from Jackson Laboratories (stock number 000671; Bar Harbor) and allowed to acclimate in the same room as the D2.*mdx* mice for 1 week before they were killed. As shown in the Results, the discrimination index of these WT mice was similar to previous work from our group (Hayward et al., 2022). Mice were housed in standard 12 h–12 h light–dark cycles and were allowed access to standard rodent chow and water ad libitum. Four days before the end of the protocol, all groups underwent novel object recognition (NOR) acclimation and testing as described below.

Mice were anaesthetized under 5% isoflurane vaporized in medical air (21% oxygen) at a flow rate of 2 L/min, then maintained at 2%–3% before exsanguination. Muscles were removed and used for another investigation (under review at the time of this publication). The right and left hippocampus were then quickly dissected from the brain, with a portion placed immediately into ice‐cold BIOPS, containing (mM): 50 MES hydrate, 7.23 K_2_EGTA, 2.77 CaK_2_EGTA, 20 imidazole, 0.5 dithiothreitol, 20 taurine, 5.77 ATP, 15 PCr and 6.56 MgCl_2_.6H_2_O (pH 7.1), or flash‐frozen in liquid nitrogen and stored at −80°C for RNA isolation and western blotting.

All experiments and procedures were approved by the Animal Care Committee at York University (AUP approval number 2016‐18) in accordance with the Canadian Council on Animal Care.

### 2.2 Novel object recognition test

The NOR test was performed as previously described (Bagdatlioglu et al., 2020; Denninger et al., 2018; Hayward et al., 2022; Leger et al., 2013). Briefly, animals were placed in an open field arena (40 cm × 40 cm × 40 cm). Sessions were recorded with a cell‐phone video camera secured above the apparatus. Testing was performed in three stages: acclimation, habituation and testing. During acclimation, mice were placed into the arena and allowed to move freely for 5 min each day for 4 days preceding testing. The next day, habituation was performed, whereby two identical objects (object 1) were placed in opposite corners of the arena, and mice were left to explore for 10 min. Objects were similar in size to the animals and were chosen to ensure novelty in all trials. After a habituation period, mice were returned to a neutral cage for 30 min. Thereafter, mice were placed back in the arena with one familiar object and one novel object (object 2) and allowed to explore for 10 min. Object exploration time was defined as the time the mouse interacted with the object, defined by sniffing or touching the object when the mouse is <2 cm from the object. Sitting or standing on the object was not included unless the mouse sniffed the object while climbing on it. For the trial to be considered valid, the animal must have interacted with the object for >20 s. The discrimination index was calculated as follows: [Time (object 2) − time (object 1)]/total time.

### 2.3 High‐resolution respirometry

Mitochondrial oxygen consumption (respiration) was measured using the in situ brain permeabilization previously described (Herbst & Holloway, 2015), with some modification. After weighing, a portion of the hippocampal samples was quickly minced with scissors in a chilled tube containing BIOPS buffer followed by immediate placement into an Oxygraph‐2K respirometer (Oroboros Instruments, Austria) containing MiR05 respiration medium (0.5 mM EGTA, 3 mM MgCl_2_, 10 mM KH_2_PO_4_, 20 mM HEPES, 60 mM potassium lactobionate, 110 mM sucrose and 1 g/L bovine serum albumin; pH 7.2) at 37°C, with constant stirring at 750 r.p.m. Samples were equilibrated in respiration buffer for 10 min before the addition of 50 μg/mL saponin to facilitate permeabilization of the tissue. After permeabilization, pyruvate‐stimulated respiration was examined in the brain using 5 mM pyruvate and 2 mM malate to generate NADH and saturate electron entry into complex I. To examine state III respiration as an index of oxidative phosphorylation, ADP was then added at a concentration of 15 μM to approximate the concentrations reported in human brains using non‐invasive magnetic resonance spectroscopy assessments (Roth & Weiner, 1991). Cytochrome *c* was added as a test of mitochondrial outer membrane integrity. All experiments demonstrated <10% increase in respiration. Each protocol was initiated with a starting [O_2_] of ∼350 μM and was completed before the oxygraph chamber [O_2_] reached 150 μM, as done previously (Perry et al., 2011; Ydfors et al., 2016). Polarographic oxygen measurements were acquired in 2 s intervals, with the rate of respiration derived from 40 data points and expressed as picomoles per second per milligram wet weight. Chemicals and reagents were purchased from Sigma (St Louis, MO, USA) or BioShop (Burlington, ON, Canada).

### 2.4 RNA isolation and quantitative PCR

Total RNA was isolated from the hippocampus using the Aurum Total RNA Mini Kit (Bio‐Rad, Mississauga, ON, Canada) according to the manufacturer's instructions, and reverse transcribed into complementary DNA by M‐MLV reverse transcriptase and oligo(dT) primers (Qiagen). Complementary DNA was then amplified in a CFX384 Touch Real‐Time PCR Detection System (Bio‐Rad) with a SYBR Green master mix and specific primers. Gene expression was normalized to an *Rplp0* control (Mele et al., 2019), and relative differences were determined using the ΔΔ*Ct* method (Livak & Schmittgen, 2001), normalized to D2A expression. The primers used to probe for mouse cytokines were as follows: interleukin‐1 beta (*IL‐1β*) forward 5′‐GCAGCACATCAACAAGAG‐3′, reverse 5′‐AGCAGGTTATCATCATCATC‐3′; tumor necrosis factor alpha (*TNF‐α*) forward 5′‐AGAATGAGGCTGGATAAGAT‐3′, reverse 5′‐GAGGCAACAAGGTAGAGA‐3′; interleukin‐6 (*IL‐6*) forward 5′‐ACAGAAGGAGTGGCTAAG‐3′, reverse 5′‐AGAGAACAACATAAGTCAGATAC‐3′; interleukin‐10 (*IL‐10*) forward 5′‐ATAACTGCACCCACTTCCCA‐3′, reverse 5′‐GGGCATCACTTCTACCAGGT‐3′; and *Rplp0* forward 5′‐TTGGAGTGACATCGTCTT‐3′, reverse 5′‐ATCTTGAGGAAGTAGTTGGA‐3′.

### 2.5 Cytokine profiling

Protein contents of TGF‐β, TNF‐α, IL‐1β, IL‐6 and IL‐10 in serum were measured by flow cytometry using the LEGENDplex Mouse Custom Panel (BioLegend, San Diego, CA, USA). Serum, collected from all groups, was diluted fourfold in the assay buffer and loaded onto the provided assay plate in a blinded manner. The assay was completed according to the manufacturer's instructions on an Attune NxT flow cytometer (Thermo Fisher). The FCS files generated on the flow cytometer were analysed using the LEGENDplex cloud‐based analysis software.

### 2.6 Western blotting

Hippocampal tissue (collected from the same mice used in high‐resolution respirometry experiments) was homogenized in cold lysis buffer containing (mM: 20 Tris–HCl, 150 NaCl, 1 EDTA, 1 EGTA, 1% Triton X‐100, 2.5 Na_4_O_7_P_2_ and 1 Na_3_VO_4_; pH 7.0) supplemented with protease and phosphatase inhibitors (Sigma) according to the protocol established by Hughes et al. (2019). Detection of electron transport chain complex subunits was performed according to the procedure described by Hughes et al. (2019). For detection of total AMPK, p‐AMPK, p‐ULK1, p62 and LC3BII/I, proteins were transferred to a 0.2 μm low‐fluorescence polyvinylidene difluoride membrane using a Bio‐Rad Trans Blot Turbo. Membranes were then blocked in Licor Intercept Blocking buffer diluted 1:1 in PBS. Membranes were incubated with appropriate primary antibodies diluted in blocking buffer (LI‐COR, Lincoln, NE, USA) as follows: p‐AMPK (1:1000; t192 CST 2535), total AMPK (1:500; CST 2532), p‐ULK1 (ser757, the target of Raptor; 1:500; CST14202), p62 (1:1000; CST 5114) and LC3B (1:1000; CST 2775) overnight at 4°C. Membranes were washed three times for 5 min in TBS‐T and incubated at room temperature with appropriate fluorescent secondary antibody (LI‐COR). The same washing protocol was repeated, after which detection was carried out using infrared imaging (LI‐COR CLx), with quantification by densitometry (ImageJ; http://imagej.nih.gov/ij/). All images were normalized to total protein from the same membrane stained using Amido Black total protein stain (A8181; Sigma).

All remaining proteins were detected on 0.45 μm nitrocellulose and blocked with 5% non‐fat dry milk in TBS‐T. Membranes were incubated with the appropriate primary antibodies as follows: APP (BioLegend 825001), soluble APPα (IBL 11088), soluble APPβ (BioLegend 813401), BACE1 (CST 5606), ADAM10 (Abcam ab1997), p‐tau (serine 202; CST 11834), total tau (CST 4019), p‐Raptor (serine 792; CST 2083), total Raptor (CST 2280), p‐p70s6K (thr389; CST 9206S), total p70s6k (Santa Cruz SC‐230), NeuN (CST 24307) and pro‐BDNF (SC SC‐65514), diluted to 1:1000 in 5% bovine serum albumin, overnight at 4°C. After primary antibody incubation and washing, membranes were incubated in horseradish peroxidase secondary antibodies diluted 1:5000 in 1% non‐fat dry milk–TBS‐T. Membranes were washed (3 × 5 min in TBST), and protein bands were imaged using enhanced chemiluminescence (Western lightning Plus‐ELC; PerkinElmer, 105001EA) and the ChemiDoc Imaging System (Bio‐Rad). All proteins were normalized to the total protein obtained from Ponceau staining. Images were analysed via AlphaView Software (ProteinSimple).

### 2.7 Statistics

Results are expressed as means ± SD. The level of significance was set to *P* < 0.05 for all statistics. D'Agostino–Pearson normality tests (GraphPad Prism software; GraphPad, La Jolla, CA, USA) confirmed that all data were normally distributed, and one‐way ANOVAs were used with the two‐stage step‐up method of Benjamini, Krieger and Yekutieli post hoc analyses for false discovery rate corrections in multiple‐group comparisons. All reported *P*‐values are false discovery rate‐adjusted *P*‐values (traditionally termed ‘q’). A required sample size of 12 was calculated based on the exploration time results of our previous work in the D2.*mdx* mouse model (Hayward et al., 2022) using a desired power of 0.80 and α of 0.05.

## 3 RESULTS

**FIGURE 1:**
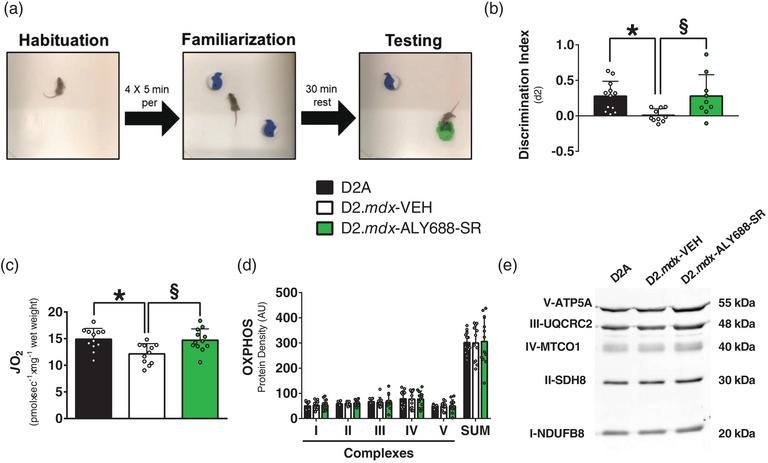
Novel object recognition (NOR) testing and hippocampal complex I‐supported respiration are preserved by ALY688‐SR in 4‐week‐old D2.*mdx* mice. (a) Representative image of the NOR task, with habituation (left), familiarization (middle) and testing (right). (b) The discrimination index was reduced in D2.*mdx*‐VEH mice and normalized with ALY688‐SR treatment. (c) State III respiration was supported by complex I substrates (NADH) pyruvate (5 mM) and malate (2 mM) and stimulated by a physiological concentration of ADP (15 μM). (d,e) Protein content of electron transport chain components was quantified in the hippocampus. Data are expressed as the mean ± SD, with *n* = 9–12 per group. ^*^ *P* ≤ 0.05 wild‐type (WT) versus D2.*mdx*‐VEH; ^§^ *P* ≤ 0.05 D2.*mdx*‐VEH versus D2.*mdx*‐ALY688‐SR.

The D2.*mdx*‐VEH mice demonstrated impaired recognition memory, as shown by a lower discrimination index in the NOR test (Figure 1a,b). This decrement was completely prevented by ALY688‐SR (Figure 1a,b). Hippocampal mitochondrial pyruvate‐supported respiration stimulated by ADP at physiological concentrations (see Materials and Methods) was lower in D2.*mdx*‐VEH but completely preserved by ALY688‐SR (Figure 1c). These differences between groups are likely reflect adaptive reprogramming intrinsic to mitochondria, given that electron transport chain subunit contents were similar in all groups (Figure 1d,e).

**FIGURE 2:**
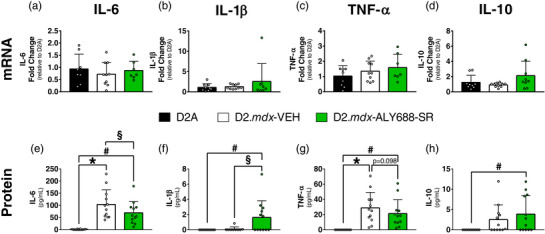
Hippocampus and serum inflammatory cytokine expression and contents. (a–d) Hippocampal mRNA fold changes of *IL‐6*, *IL‐1β*, *TNF‐α* and *IL‐10* were expressed relative to wild‐type (WT; D2A) expression. (e–h) Serum levels of IL‐6, IL‐1β, TNF‐α and IL‐10 were quantified. Data are expressed as the mean ± SD, with *n* = 7–12 per group. ^*^ *P* ≤ 0.05 WT versus D2.*mdx*‐VEH; ^#^ *P* ≤ 0.05 WT versus D2*.mdx*‐VEH; ^§^ *P* ≤ 0.05 D2.*mdx*‐VEH versus D2.*mdx*‐ALY688‐SR. Abbreviations: IL‐6, interleukin‐6; IL‐10, interleukin‐10; IL‐1β, interleukin‐1 beta; TNF‐α, tumor necrosis factor alpha.

We then assessed cytokine markers, given that the parent compound of ALY688‐SR (ALY688) has been shown to have anti‐inflammatory effects. No significant changes were observed in hippocampal mRNA of *IL‐6*, *IL‐1β*, *TNF‐α* or *IL‐10* (Figure 2a–d). Cytokine protein contents were assessed in serum owing to tissue limitations in the hippocampus. Serum IL‐6 and TNF‐α were significantly elevated in D2.*mdx*‐VEH mice versus D2A control animals (Figure 2e,g). ALY688‐SR attenuated IL‐6 (Figure 2e) and increased IL‐1β relative to D2.*mdx*‐VEH, while also increasing IL‐10 versus WT (Figure 2f,h).

**FIGURE 3:**
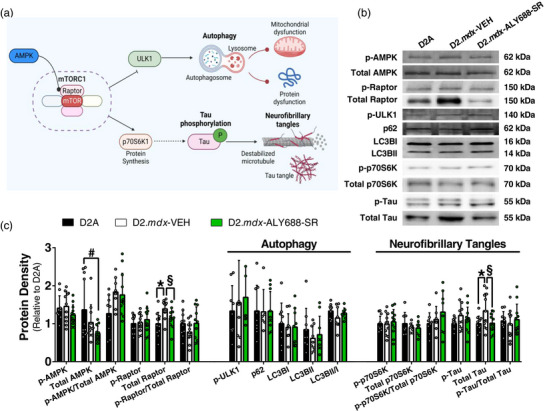
AMPK and downstream protein markers related to autophagy and neurofibrillary tangles. (a) Theoretical cascade linking AMPK (an adiponectin receptor target) to factors related to autophagy and neurofibrillary tangles. The AMPK target, Raptor, is rendered inactive upon its phosphorylation, allowing for induction of autophagy through phosphorylation of ULK‐1, leading to clearance of dysfunctional mitochondria and proteins (Lee et al., 2010; Norwitz & Querfurth, 2020). Alternatively, activation of the mTORC1 complex leads to increased protein synthesis via p70s6k1, which has been implicated in tau phosphorylation, destabilization of microtubules and generation of neurofibrillary tangles (Iqbal et al., 2010; Pei et al., 2006). Made with BioRender. (b,c) Western blot markers for targets outlined, assessed in hippocampal tissue (a). Data are expressed as the mean ± SD, with *n* = 6–12 per group. ^*^ *P* ≤ 0.05 WT versus D2.*mdx*‐VEH; ^§^ *P* ≤ 0.05 D2.*mdx*‐VEH versus D2.*mdx*‐ALY688‐SR. Abbreviations: AMPK, AMP kinase; mTORC1, mammalian target of rapamycin complex 1; p70s6k1, ribosomal protein S6 kinase β1; Raptor, regulatory‐associated protein of mTOR; ULK1, Unc‐51 like autophagy activating kinase; WT, wild‐type.

We next assessed markers of potential pathways linking AMPK or inflammation to neural function, given that AdipoR agonism activates AMPK signalling in various models (Abou‐Samra et al., 2020; Da Eira et al., 2020) and lowers inflammation, as noted above. As shown in Figure 3b,c, total AMPK protein was lower after treatment with ALY688‐SR. Although difficult to explain, the degree of activation (p‐AMPK or p‐AMPK/AMPK) was not altered. Given that tissues were harvested ∼20–24 h after the last injection, it is possible that AMPK activation might have occurred earlier, given that previous work showed rapid increases in AMPK phosphorylation, albeit in L6 muscle cells after 30 min of treatment (Sung et al., 2022). Alternatively, it is possible that a different drug dose would be required to see an effect on APMK phosphorylation. ALY688‐SR also completely prevented the increases in protein contents of total tau (marker of neurofibrillary tangles) and total Raptor (upstream regulator of tangles and autophagy) seen in D2.*mdx* versus WT. However, phosphorylation (both absolute and relative to total protein) of tau and Raptor (serine 792; an AMPK‐specific phosphorylation site; Gwinn et al., 2008) were similar in all groups (Figure 3a–c).

**FIGURE 4:**
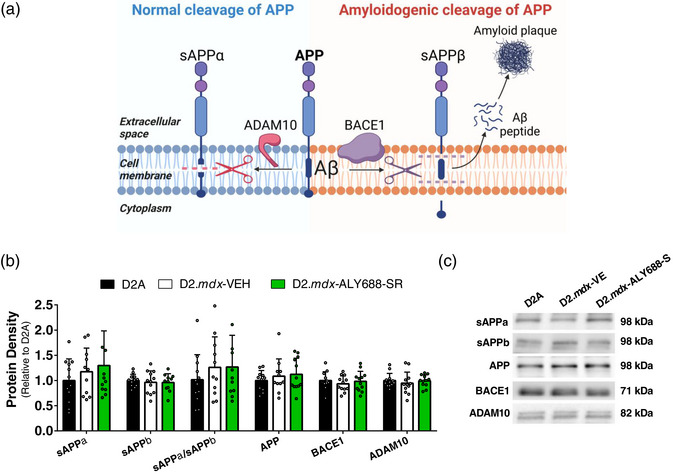
Protein markers of amyloidogenic cascade. (a) The development of neuritic plaques occurs when cleavage of amyloid precursor protein (APP) into amyloid beta (Aβ) peptides and soluble APPβ (sAPPβ) via beta‐secretase 1 (BACE1) (right) is favoured over conventional cleavage of APP (left). Adapted from Mueller et al. (2018). Made with BioRender. (b,c) Western blot markers for targets outlined, assessed in hippocampal tissue (a). Data are expressed as the mean ± SD, with *n* = 11–12 per group. Abbreviations: Aβ, amyloid beta; ADAM10, A disintegrin and metalloproteinase 10; APP, amyloid precursor protein; BACE1, beta‐secretase 1; sAPPα, soluble APP‐alpha; sAPPβ, soluble APP‐beta.

Markers of amyloidogenesis (Figure 4a–c) were similar in all groups, as were brain‐derived neurotrophic factor (BDNF) and neuronal nuclear protein (NeuN) (data not shown). ALY688‐SR did not alter body weight, tibial length, spleen mass, kidney mass or liver mass (data not shown).

## 4 DISCUSSION

Here, we show that the new AdipoR agonist, ALY688‐SR, improved recognition memory in young D2.*mdx* mice after a short‐term, 21‐day treatment protocol. This effect was associated with a complete restoration of hippocampal pyruvate‐supported mitochondrial respiration (index of glucose oxidation) when stimulating ATP synthesis with an ADP concentration matching the level reported in vivo in the human brain (Tracey et al., 1995). This finding is interesting, given the high dependence of neurons on glucose as a fuel source. Given that this protocol, in part, on complex I oxidation of NADH, the finding might also imply that complex I dysfunction occurs in the hippocampus, similar to reports in muscle from D2.*mdx* mice (Bellissimo et al., 2023; Hughes et al., 2019, 2020; Ramos et al., 2020) and in other regions of the brain in C57BL/10ScSn‐*mdx* mice (Tuon et al., 2010). Hippocampal mitochondrial relationships to NOR have also been reported previously. For example, reduced NOR and hippocampal mitochondrial structural dynamics were altered in ageing‐related neurodegeneration (Mishra & Thakur, 2022), whereas improvements in pyruvate‐supported respiration and NOR were observed in a model of erythropoietin overexpression (Jacobs et al., 2021).

The precise relationship between dystrophin mutations and mitochondrial stress in the brain is not clear but might be informed by previous discoveries in muscle from dystrophin‐deficient animal models (Bellissimo et al., 2022; Nghiem et al., 2017), which generally implicate calcium overload, redox stress and inflammation as possible links (Guiraud & Davies, 2017). Increases in serum IL‐6 and reductions in mitochondrial pyruvate oxidation in the present study align with this possibility, particularly given that ALY688‐SR partly attenuated IL‐6 while completely preserving respiration and recognition memory. Although hippocampal inflammatory markers were unchanged, the reductions in serum IL‐6 with ALY688‐SR present the possibility that systemic inflammation caused by the well‐characterized muscle damage in D2.*mdx* mice (Bellissimo et al., 2023; Coley et al., 2016; Fukada et al., 2010; Hughes et al., 2019) might contribute a trigger separate from the direct effects of the dystrophin mutation in the hippocampus. Indeed, pharmacological inhibition of IL‐6 in the hippocampus improved long‐term memory in rats (Balasubramanian et al., 2022), but the effects on mitochondrial bioenergetics were not examined.

Improved recognition memory was also related to increased contents of tau protein and the inhibitory complex of mTOR, Raptor, both of which are downstream of AdipoR signalling (Domise et al., 2016; Gwinn et al., 2008). The lack of change in their phosphorylation state does not necessarily rule out a potential role for these pathways in mediating the disease or treatment effects, given that the tissues were harvested 20–24 h after the last dose. Assessing the activity of these and other signalling cascades shortly after dosing might provide more insight into their potential contributions. This timing consideration might also explain why AMPK itself was not phosphorylated in response to the drug despite being a known downstream target of AdipoR (Yamauchi et al., 2002; Zhang et al., 2022) and considering that both AMPK and p38MAPK are phosphorylated after 30 min of ALY688 treatment in L6 muscle cells (Sung et al., 2022). Future experiments could extend these findings to incubations in neurons.

### 4.1 Limitations and future directions

In addition to the hippocampus, the NOR test is dependent on multiple regions of the brain not investigated in the present study, such as the prefrontal cortex. Although we were unable to obtain sufficient sample of this region, given the small size of the brain at 4 weeks of age, prior research has shown that greater markers of amyloidogenesis occur in this region compared to only one marker in the hippocampus at 8 weeks of age in D2.*mdx* mice (Hayward et al., 2022). The present study found no changes in any such marker in the hippocampus, which might be related to the younger age, and we cannot rule out the possibility that reduced NOR in D2.*mdx*‐VEH was related to such markers in the prefrontal cortex.

Tissue limitations also restricted analyses of mechanisms underlying the mitochondrial responses noted herein, but future studies could consider a variety of mechanisms not limited to post‐translational modifications of pyruvate dehydrogenase (noting the use of pyruvate as a substrate in our respirometric protocol) in addition to alterations to mitochondrial morphology‐linked changes in the regulation of oxidative phosphorylation and global mitochondrial content measures beyond the OXPHOS markers that were assessed. Also, the lack of change in protein markers of autophagy and neurotrophic factors does not reflect the activity of these pathways, which require assessments of their kinetic aspects with additional methodologies. As such, these processes could still be considered in future research with respect to how dystrophin mutations impair recognition memory and how ALY688‐SR restores this cognitive function, given that AdipoRs and/or AMPK (a target of AdipoR) have been linked to autophagy, neurofibrillary tangles or amyloidogenesis (Chan et al., 2012; He et al., 2021; Pei et al., 2006; Zhang et al., 2011). Lastly, the relative contributions of direct effects of ALY688‐SR on brain AdipoRs (Clain et al., 2022; Thundyil et al., 2012) or indirect effects remain to be determined.

## 5 CONCLUSION

ALY688‐SR prevented impairments in recognition memory after 21 days of treatment in young D2.*mdx* mice. Given that there is an unmet need for therapies that improve memory and other cognitive functions in DMD, these findings provide a foundation for continued exploration into the mechanisms and potential of adiponectin receptor agonism in treating cognitive dysfunction separately from the myopathy in this debilitating disease.

## AUTHOR CONTRIBUTIONS

Study design and conception were prepared by Catherine A. Bellissimo, Laura N. Castellani, Shivam Gandhi, Gary Sweeney, Christopher G. R. Perry and Allysta Pharmaceuticals, and all authors contributed to the rationale for specific measurements. Data acquisition, analysis and interpretation of the work were performed by all authors. The manuscript was written by Catherine A. Bellissimo and Christopher G. R. Perry. All authors contributed to the manuscript, approved the final version and agree to be accountable for all aspects of the work in ensuring that questions related to the accuracy or integrity of any part of the work are appropriately investigated and resolved. All persons designated as authors qualify for authorship, and all those who qualify for authorship are listed.

## CONFLICT OF INTEREST

This study was funded, in part, by Allysta Pharmaceuticals. G.S. is a Scientific Advisor for Allysta Pharmaceuticals.

## Data Availability

Data can be made available upon reasonable request to the corresponding author.
